# ADME gene polymorphisms do not influence the pharmacokinetics of docetaxel: Results from a population pharmacokinetic study in Indian cancer patients

**DOI:** 10.1002/cam4.4026

**Published:** 2021-06-22

**Authors:** Anand Patil, Bharati Shriyan, Parsshava Mehta, Mrudula Patil, Murari Gurjar, Manjunath Nookala, Vijay Patil, Amit Joshi, Vanita Noronha, Kumar Prabhash, Vikram Gota

**Affiliations:** ^1^ Department of Clinical Pharmacology Advanced Centre for Treatment, Research and Education in Cancer Tata Memorial Centre Mumbai India; ^2^ Homi Bhabha National Institute Mumbai India; ^3^ Department of Medical Oncology Tata Memorial Hospital Mumbai India

**Keywords:** ADME, docetaxel, pharmacogenetics, population pharmacokinetics

## Abstract

**Background:**

Pharmacokinetics (PK) of docetaxel is characterized by high inter‐individual variability (IIV). While covariate models that explain the PK variability of docetaxel exist, not much is known about the effects of genetic variations on docetaxel disposition.

**Methods:**

Fifty patients with head and neck or prostate cancer were enrolled of whom two patients withdrew consent before the start of the study. Docetaxel was administered at either 50 or 75 mg/m^2^ as intravenous infusion over 1 h. One pharmacogenetic sample and a series of PK samples, either intensive (*N* = 5; 13 samples each) or sparse (*N* = 43; 6 samples each), were collected from each patient. Docetaxel levels were estimated using a validated HPLC method. Polymorphic loci on the Absorption, Distribution, Metabolism, and Elimination (ADME) genes were identified using the PharmacoScan array platform. Population pharmacokinetic analysis was carried out using NONMEM v7.2.

**Results:**

Docetaxel PK was well characterized by a three‐compartment model. Clearance (Cl) was found to be 18 L/h with an IIV of 45.3%. None of the genetic variants showed significant covariate effect on the Cl of docetaxel. Patients with abnormal alanine aminotransferase (ALT) were found to have 25% lower Cl as compared to patients with normal ALT values. However, the covariate effect could not be established in the final model possibly due to lack of adequate number of patients with abnormal ALT.

**Conclusion:**

Genetic polymorphisms in the ADME gene do not explain the IIV in PK of docetaxel. However, patients with abnormal liver function might require dose reduction.

Clinical trial registration: Not applicable since participants in this study received treatment that was standard of care.

## Graphical abstract

The study aims to explain the inter‐individual variability in the pharmacokinetics of docetaxel using a population pharmacokinetic approach. Our results conclusively exclude pharmacogenetic variants as a significant covariate, while recommending dose modification for patients with hepatic impairment.

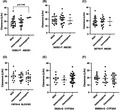

## 1 INTRODUCTION

Docetaxel, a tubulin‐interacting agent, is a widely used chemotherapeutic agent for nearly 25 years. It is approved for the treatment of head and neck cancers along with gastric, ovarian, lung and breast cancers. Docetaxel is typically administered in the range of 60–100 mg/m^2^ for several indications.^1^ However, dosing practices are not uniform across the globe. For instance, in Japan, doses recommended are typically 25% less than the dose used in the Caucasian population. This is because the Japanese were found to be more susceptible to toxicity than their Caucasian counterparts though the reasons behind these still needs to be elucidated, pharmacogenetics could be one of the reasons for this difference.^2^

Docetaxel pharmacokinetics (PK) is marked by large inter‐individual variability (IIV) which predominantly accounts for the difference in toxicity and efficacy between individuals.^3^, ^4^, ^5^ Part of this IIV in PK can be explained by the nonsynonymous germline variants particularly in the genes encoding the drug metabolizing enzymes or the drug transporters. Identifying the variants in these genes responsible for the drug's Absorption, Distribution, Metabolism, and Elimination (ADME) pave the way for a more personalized approach toward drug dosing.

Docetaxel metabolism mainly takes place in the liver by CYP3A4 and CYP3A5 into four different metabolites.^6^ The limited activity of all metabolites suggest that docetaxel metabolism contributes to its inactivation.^7^ Docetaxel is a known substrate of the largest family of drug transporters, the solute carriers (SLC), which account for 48% of the total number of transporters.^8^ The SLC family is also responsible for hepatocellular uptake of docetaxel from the sinusoidal blood. The elimination of docetaxel and its metabolites is facilitated by the ABCB1 and ABCC2 transporters,^9^ which are also responsible for secretion of docetaxel from the liver into the bile. Certain polymorphisms in CYP3A and ABCB1 genes are known to affect docetaxel disposition. For instance, CYP3A4*1B is associated with increased clearance (Cl) of docetaxel.^10^, ^11^

The currently used dosing strategy based on body surface area (BSA) does not reduce the IIV, markedly underscoring the importance of other variables.^8^ Potential factors contributing to the variability in PK are etiology and severity of the cancer being treated along with any unintended drug interactions and impaired hepatic and renal functions.^10^ Despite these variables being potentially significant, unexplained variability in PK due to ADME gene polymorphisms can still have a significant impact on the efficacy and toxicity of the drug.

Our study aimed at identifying such covariates that significantly affect docetaxel disposition based on which individualized dosing approaches could be adopted.

## 2 PATIENTS AND METHODS

### 2.1 Patients and treatment

Patients aged 18 years and above with cytologically and histologically proven solid malignancy, for which docetaxel was prescribed, were enrolled in the study. The enrolled participants were required to have adequate hematologic (hemoglobin ≥8 g/dl), hepatic (bilirubin ≤2 times the upper limit of normal (ULN) and, AST and ALT ≤3 times the ULN) and renal (creatinine clearance [CrCl] ≥50 ml/min) function with an ECOG PS of 0–2. Patients with serious comorbid conditions, including severe cachexia (weight loss >10% of the body weight in the past 6 months), which can affect the disposition of docetaxel, or any cytochrome inhibitor or inducer 14 days preceding docetaxel infusion were excluded. The dose of docetaxel administered was 50–75 mg/m^2^, infused over 1 h according to the local protocol for standard care. All patients received standard premedication that comprised of aprepitant (125 mg on day 1 and 80 mg on day 2 PO); a 2‐day regimen of granisetron 1 mg I.V.; a similar 2‐day regimen of ranitidine and dexamethasone at 50 mg and 8 mg I.V. respectively. Promethazine (12.5 mg I.V.) was administered a day before starting docetaxel. Other oral premedications included a 3‐day dexamethasone (8 mg OD) and ranitidine (150 mg BD) regimen from day 2 to day 4. Prophylactic granulocyte colony stimulating factor (G‐CSF) was used to alleviate the risk of any hematological toxicities.

### 2.2 Ethics statement

The study was approved by the Institutional Ethics Committee of Tata hospital. All trial participants provided written informed consent prior to their enrolment. The study was carried out in accordance with the Declaration of Helsinki and International Conference on Harmonization—Good Clinical Practice (ICH‐GCP) guidelines.

### 2.3 Pharmacokinetic sampling

Out of the 50 subjects enrolled in the study, two patients withdrew consent before the first pharmacokinetic sample was collected. Thus, 48 subjects were available for the final analysis. Out of the 48 subjects, 5 subjects were enrolled for traditional sampling comprising of 13 sampling points. The traditional sampling was a rich sampling strategy carried out to characterize the complete pharmacokinetic profile of docetaxel. Samples were collected at 0.5 h, end of infusion, 1.085, 1.17, 1.25, 1.5, 2.0, 2.5, 3.0, 4.0, 6.0, 8.0 and 12.0 h with respect to start of infusion. The remaining 43 patients were enrolled for sparse sampling, where a total of 6 samples were collected at 0.5 h, end of infusion, 1.17, 1.5–2.0, 3.0–4.0 and 6.0–12.0 h. An additional sample for pharmacogenetic analysis was collected from each subject in 3 ml EDTA vacutainers. The PK blood samples were centrifuged at 1008 *g* for 10 min. The supernatant plasma was collected and stored in pre‐labelled 1.7 ml microcentrifuge tubes at −20℃ pending further analysis. Docetaxel levels were determined with a validated high‐performance liquid chromatography method as described in Andersen et al.^12^ The method was validated over a linear range from 50 to 5000 ng/ml.

### 2.4 Pharmacogenetic analysis

#### 2.4.1 DNA extraction

Genomic DNA was extracted from a 3 ml EDTA blood sample using the QiAmp DNA isolation kit (Qiagen, GmBH) according to the manufacturer's instructions.

#### 2.4.2 PharmacoScan array

In line with the manufacturer's instructions, genomic DNA was amplified (amplification and multiplex PCR). These amplified products were then fragmented, pooled, resuspended, and hybridized to the PharmacoScan Array platform (Thermo Fisher Scientific). Arrays are scanned on the GeneTitan Multi‐Channel Instrument (Thermo Fisher Scientific). The generated data were analyzed using the Applied Biosystems Axiom^™^ Analysis Suite software (Thermo Fisher Scientific).

### 2.5 Population pharmacokinetic and pharmacogenetic analysis

The Population pharmacokinetic (PopPK) analysis was conducted using a non‐linear mixed‐effects modeling program (NONMEM, v 7.2; Icon Development Solutions). The interface used to run NONMEM was Pirana (v 2.8.1). R (v 3.4.2) was used to perform data management and wrangling. The diagnostic plots were generated using the *xpose* (v 4.6.1) and *vpc* (v 1.1.0) package within R.

The interindividual and IIV applied was estimated using first‐order conditional estimation with interaction (FOCEI). The covariance step in NONMEM was calculated, to evaluate the precision of the variable estimates. The three‐compartment structural model was a better fit than the two‐compartment model, similar to the previously described base model by Bruno et al.^4^ Various error models such as the exponential and additive models were compared for IIV on pharmacokinetic parameters. The additive, proportional, and mixed (additive and proportional) error models for the residual variability among plasma concentrations were compared. The influence of each covariate was evaluated by the difference in objective function value (OBJ), between the base model and the model involving the covariate by stepwise forward inclusion and backward elimination methods. In the forward inclusion method, a *p*‐value <0.05 (ΔOBJ > 3.841 with one degree of freedom assuming a chi‐squared distribution), and in the backward elimination method for a *p*‐value of <0.005 (ΔOBJ > 7.88), were considered statistically significant.

From the total data set, 6 observations from a single patient were excluded because it failed to fit into the model. Another patient was excluded from the analysis since the pharmacogenetic information was unavailable. The biologically plausible covariates evaluated were age, sex, serum creatinine (SrCr), albumin (Alb), alanine aminotransferase (ALT), aspartate aminotransferase (AST), alkaline phosphatase (ALP), and CrCl. The Cockcroft and Gault equation was used to calculate the creatinine Cl.^13^

A covariate analysis, using the following model, was performed to assess the influence of these factors on the CL of docetaxel. The continuous variables were tested using both the continuous (Equation 1) and categorical (Equation 2) approach. The covariates were tested univariately on CL.

(1) $$\text{Cl}={\text{CL}}_{\text{pop}}\times {\left(\text{COV}/\text{M - COV}\right)}^{\theta_{x}},$$

where CL_pop_ is the population typical value, COV is the respective covariate value, M‐COV the median value of the respective covariate and *θ_x_* is the covariate coefficient.

(2) $$\text{Cl}={\text{CL}}_{\text{pop}}\times \theta_{x}^{\text{cov}},$$

where CL_pop_ is the population typical value and *θ_x_* the fractional change to be estimated for the respective COV (covariate). The COV takes a binary value of 0 or 1. For age above 60 (geriatrics) the COV equaled 1 and 0 otherwise. In the case of sex, males take a value of 0 and females a value of 1. In the case of SrCr, AST, ALT, and ALP if the values were above the ULN for our hospital, the COV equaled at 1 and 0 otherwise. In case of Alb values below the normal range for our hospital the COV equaled 1 and 0 otherwise. The COV effect of CrCl was calculated using equation 3 where patients with normal renal functions (CrCl >90 ml/min) were grouped as 0, mild kidney damage (CrCl 60–89 ml/min) were grouped as 1 and moderate renal damage (CrCl 30–59 ml/min) were grouped as 2.

(3) $$\text{CL}={\text{CL}}_{\text{pop}}\times \theta_{x}^{\text{mild}}\times \theta_{y}^{\text{moderate}}.$$

The effect of pharmacogenetic variants of cytochrome P450 enzymes and drug transporters such as CYP3A4 (A392G), CYP3A5 (A6986G), SLCO1B1 (G1187A) and ABCB1 (C1236T, G2677T, C3435T) were examined. A separate fixed effect was constituted for fractional changes in the different genotypes (wild type, heterozygous and homozygous mutants). For example, a change in Cl of docetaxel due to C1236 ABCB1 carriers was described as follows:

(4) $$\text{CL}={\text{CL}}_{\text{pop}}\times \theta_{x}^{\text{heterozygous}}\times \theta_{y}^{\text{homozygous}},$$

where CL_pop_ is the population typical value of Cl for the wild type, *θ_x_* and *θ_y_* are the fractional changes for heterozygous and homozygous carriers of C1236 ABCB1 polymorphism respectively, on the Cl of docetaxel.

### 2.6 Model validation

The diagnostic plots used to evaluate the model were as follows: observed (OBS) versus population predicted (PRED) and individual predicted (IPRED), and conditional weighted residuals (CWRES) versus time to identify bias corresponding to model misspecification. The empirical Bayes‐estimate diagnostic method was used to evaluate eta shrinkage.^14^ The original dataset was used to generate a total of 1100 replicate datasets by using Monte Carlo simulations in NONMEM to perform prediction‐corrected visual predictive check (pcVPC). The pcVPC were used to evaluate the performance of the final PopPK model for docetaxel. The original dataset was used to generate a total of 1100 replicate datasets for the bootstrap analysis. The model validation was performed using the bootstrap procedure to obtain 95% CI of the estimates and comparing them to the original dataset.

## 3 RESULTS

### 3.1 Patient characteristics

A total of 50 patients were screened and enrolled in the study. Two patients withdrew consent before starting the study. Thus, 48 patients were available for analysis. Forty‐three out of 48 subjects who participated in the study were males, and the most common tumor type was head and neck cancer (41 of the 48 patients, 85.41%). Patients received one of the five most commonly available docetaxel brands in the pharmacy. Majority of the patients received the brand Doceaqualip (Intas Pharmaceuticals; *n* = 23) followed by Docetere (Dr. Reddy's Laboratories; *n* = 20), Zytax (Zydus Cadila; *n* = 3) and one patient each received the brands Taxotere (Sanofi‐Aventis) and Docetax (Cipla). A summary of demographics for the 48 patients is given in Table 1. Further, two patients were excluded from the final analysis for reasons mentioned in Section 2.5.

**TABLE 1:** Baseline characteristics of patients

Patient demographics	*n* = 48
Gender	
Male	43 (89.6%)
Female	5 (10.4%)
Type of cancer	
Head and neck cancer	41 (85.41%)
Prostate cancer	7 (14.59%)

	Median (range)
Age, years	44 (24–79)
Body surface area, m^2^	1.64 (1.1–2.0)
Dose, mg	120 (65–150)
Serum creatinine, mg/dl	0.9 (0.6–1.4)
Creatinine clearance, ml/min	86.55 (32.89–131.5)
Albumin, g/dl	4.1 (2.8–5.2)
Aspartate transaminase, IU/L	22 (11–57)
Alanine transaminase, IU/L	26 (9–90)
Alkaline phosphatase, IU/L	113 (55–382)
Serum bilirubin, mg/dl	0.58 (0.39–1.3)

### 3.2 PopPK analysis of docetaxel

The observed plasma concentrations‐time profile was well predicted by the previously developed three‐compartment structural pharmacokinetic model of Bruno et al.^4^ A drop in OBJ value of −43.21 points (−758.44 vs. −715.23) for the three‐compartment model as compared to the two‐compartment model better described the docetaxel plasma concentration. The estimated pharmacokinetic parameters were Cl (L/h), inter‐compartment Cl (Q_2_ and Q_3_, L/h), and the central and peripheral volumes of distribution (V_1_, V_2_ and V_3_, L). The random effect parameters for IIV on Cl, Q_3_, and V_3_ was explained by the exponential error model, and the residual variability was explained by a proportional error model which provided a better fit to the data compared to other error models. The final estimates of the population parameters are shown in Table 2. Cl showed a high IIV of 45% which is as per previously published literature.^3^, ^4^, ^5^

**TABLE 2:** Population pharmacokinetic parameters of docetaxel

Pharmacokinetic parameters	Estimate of original dataset (RSE, %)	Median of bootstrap replicates (2.5–97.5% CI), *N* = 1100
Clearance (L/h)	18 (20)	17.59 (6.25–23.07)
Volume of compartment 1 (L)	5.8 (10)	5.66 (2.90–6.86)
Volume of compartment 2 (L)	2.65 (15)	2.79 (1.97–4.58)
Volume of compartment 3 (L)	468 (28)	478.8 (290.36–1045.70)
Inter‐compartmental clearance 1–2 (L/h)	6.49 (37)	6.74 (3.44–28.32)
Inter‐compartmental clearance 1–3 (L/h)	29.7 (14)	30.20 (23.08–42.13)
Inter‐patient variability		
Clearance (%)	45.3 (20)	45.8 (29.84–83.27)
Inter‐compartmental clearance 1–3 (%)	53.5 (10)	51.9 (41.14–63.13)
Volume of compartment 3 (%)	78.5 (17)	75.42 (44.95–101.56)
Residual variability		
Proportional error (%)	30.93 (5)	30.6 (27.71–33.83)

Abbreviations: CI, confidence interval; RSE, relative standard error.

#### 3.2.1 Covariate modeling

**FIGURE 1:**
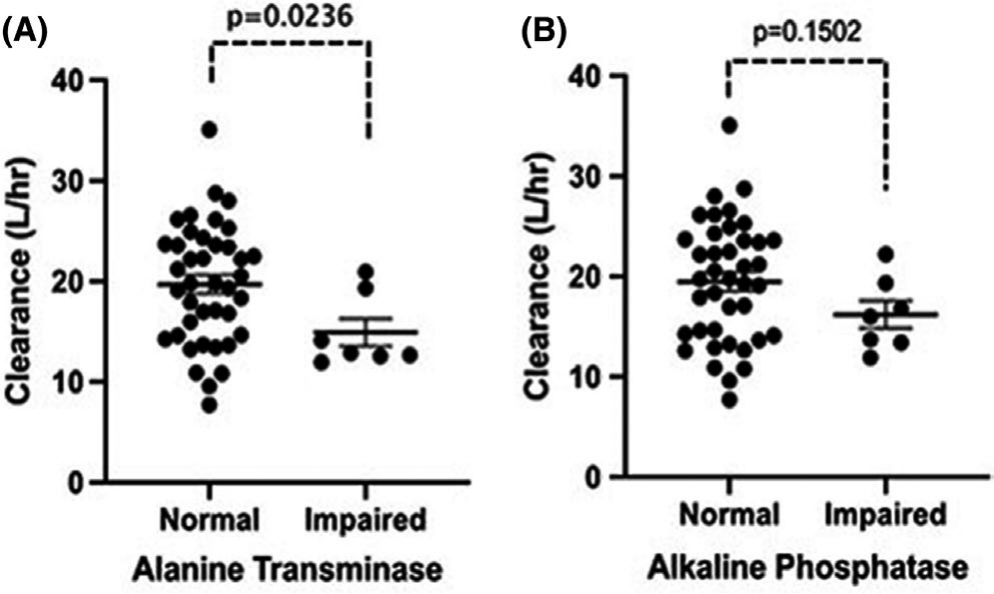
Effect of hepatic impairment on clearance of docetaxel. (A) Normal and elevated levels of alanine transaminase versus clearance (L/h) and (B) normal and elevated levels of alkaline phosphatase versus clearance (L/h). The *bar* shows the mean and standard error in each group

The covariates were tested both in a continuous and categorical approach. In the continuous approach, after forward inclusion‐backward elimination only ALP had a drop in OBJ (ΔOBJ = 5.306; *p* = 0.02). Thus, it was not significant in the backward elimination and hence not included. The full covariate model proposed by Bruno et al. was not included since the hepatic functions, which are potential covariates for the Cl of docetaxel, fell within the normal range for most patients. The covariates were also tested in a categorical approach since very few patients had abnormal levels and hence did not offer sufficient spread in the data. Nevertheless, patients with abnormal ALT were found to have 25% lower Cl as compared to patients with normal ALT values as shown in Figure 1A (Cl vs. ALT, *p* = 0.04, ΔOBJ = 3.964). Patients with abnormal ALP had a similar trend as seen with abnormal ALT, although the covariate effect was not statistically significant (Cl vs. ALP, *p* = 0.15) (Figure 1B). Other covariates tested on the Cl of docetaxel had no significant effect.

**FIGURE 2:**
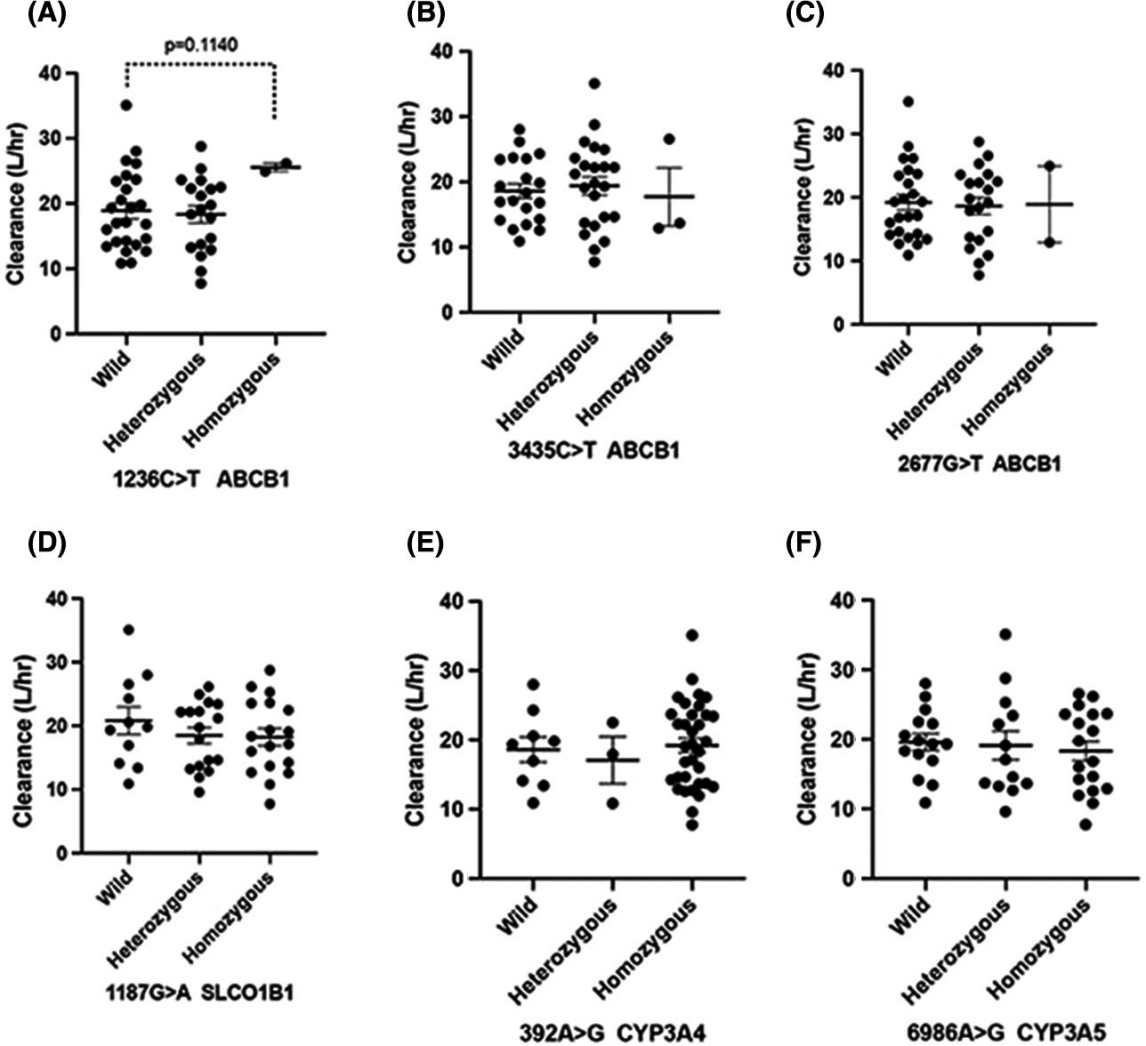
Pharmacogenetic effect on the clearance of docetaxel. Effect of genetic polymorphism of ABCB1 1236C>T, 3435C>T, 2677G>T (A–C); SLCO1B1 1187G>A (D); CYP3A4 392A>G (E); CYP3A5 6986 A>G (F); on the predicted individual docetaxel clearance (L/h). The *bar* shows the mean and standard error in each group

The PopPK model was evaluated based on our hypothesis that polymorphisms could influence the PK of docetaxel. In a bid to identify the effect of genetic covariates on Cl, polymorphisms in the genes responsible for both transportation (ABCB1, ABCC2, and SCLO1B1) and metabolism (CYP3A4 and CPY3A5) of docetaxel did not have any impact as shown in Figure 2 and Table 3.

**TABLE 3:** Stepwise covariate analysis of the genetic polymorphisms

Polymorphism	Pharmacokinetic variable	OFV	*p*‐value
Base model		−758.44	
CYP3A4*1B	Cl (L/h)	−758.78	0.84
CPP3A5*3	Cl (L/h)	−759.364	0.63
ABCBI			
C1236GT	Cl (L/h)	−760.986	0.28
	V3 (L)	−760.656	0.33
C3435T	Cl (L/h)	−758.58	0.93
	V3 (L)	−761.58	0.20
G2677T	Cl (L/h)	−758.69	0.88
	V3 (L)	−761.85	0.18
SLCOIB1			
G1187A	Cl (L/h)	−759.78	0.51
	V3 (L)	−760.20	0.41

Abbreviation: OFV, objective function value.

### 3.3 Model validation

**FIGURE 3:**
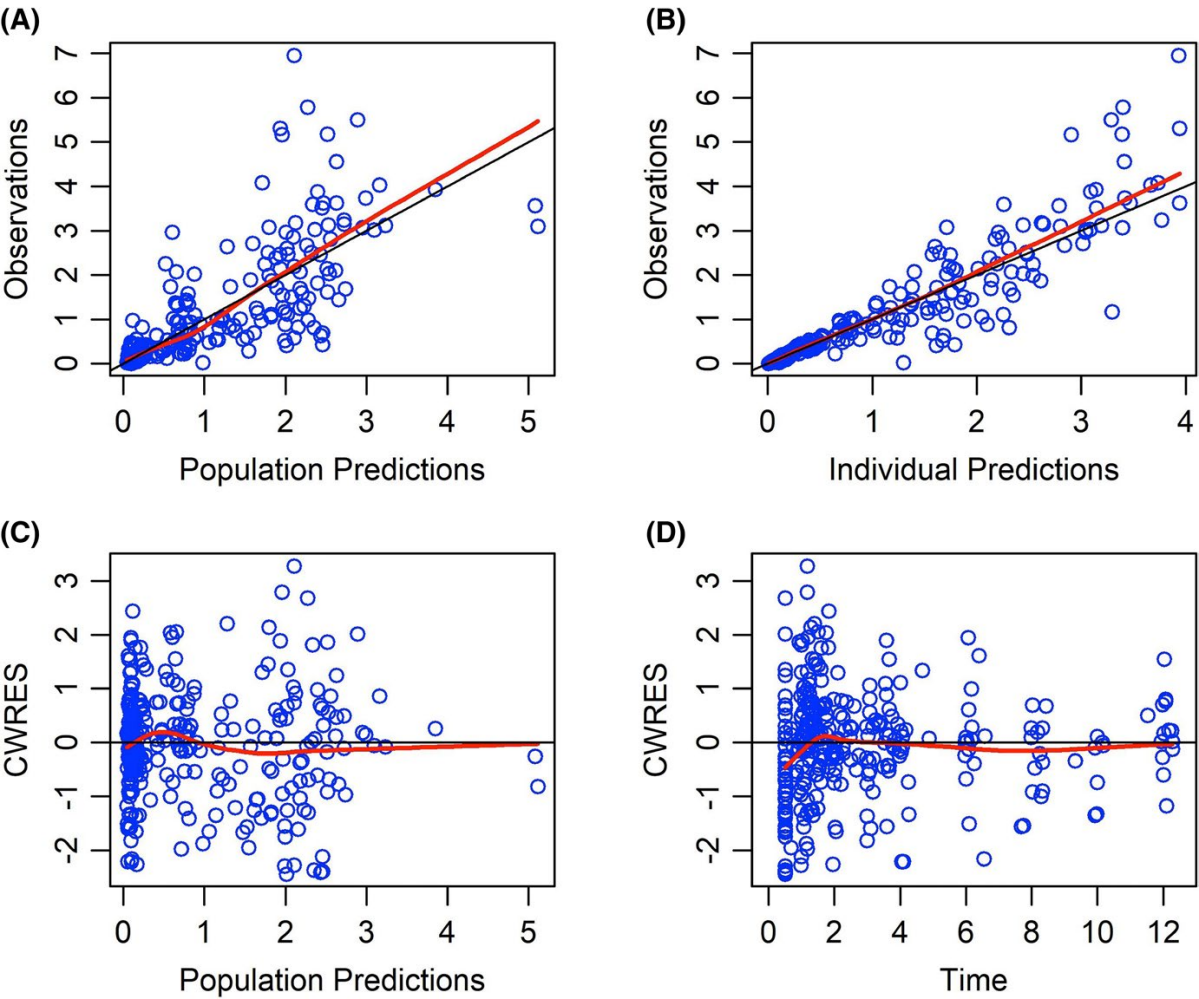
Goodness of fit plots for the final population model of docetaxel. (A) Population predicted concentration (mg/L) or (B) individual predicted concentration (mg/L) versus observed concentrations (mg/L), (C) population predicted concentrations (mg/L) versus conditional weighted residuals (CWRES) (D) time (h) versus conditional weighted residuals

**FIGURE 4:**
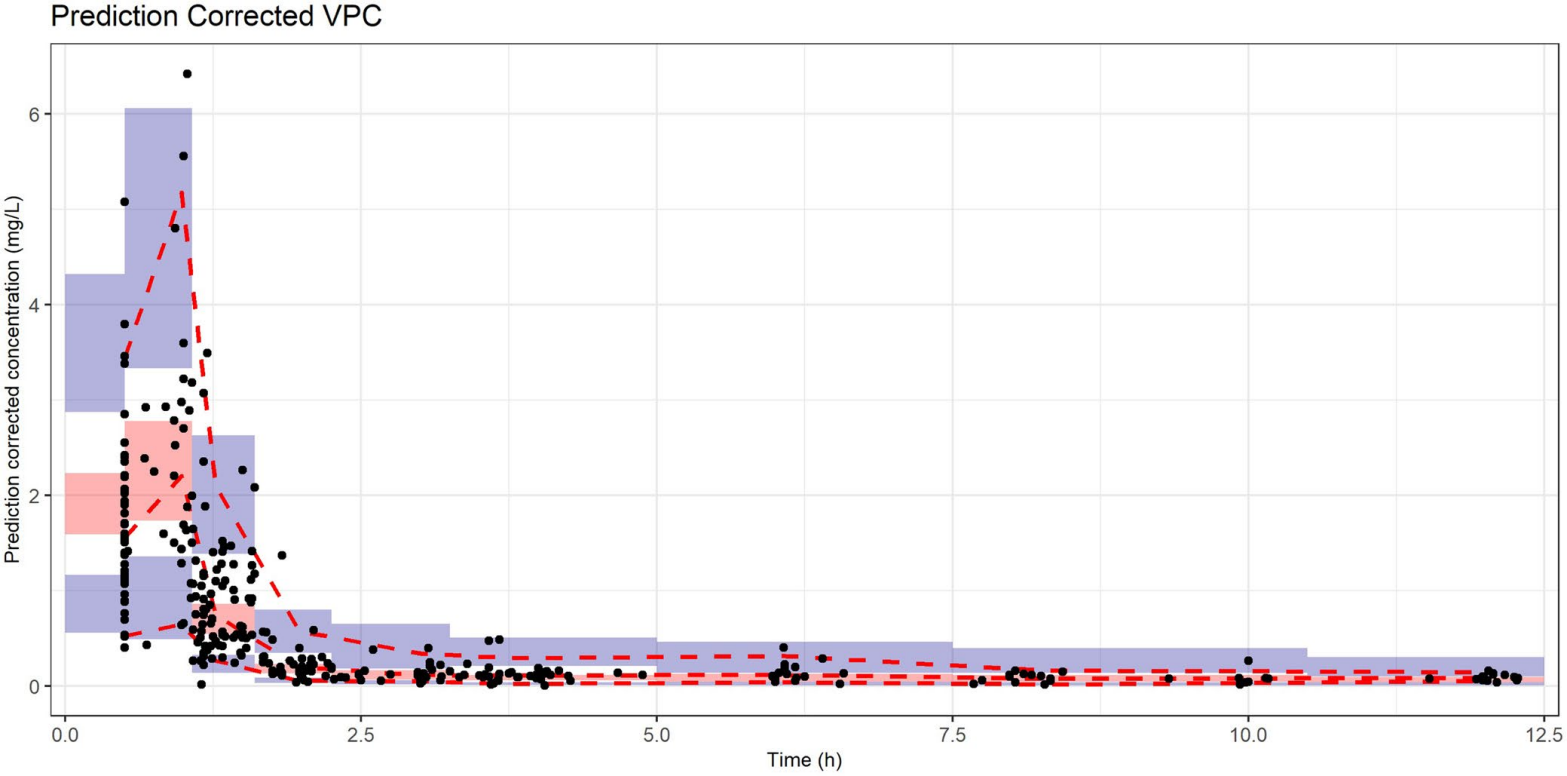
Prediction corrected visual predictive check (VPC). The black dots represent the prediction corrected observations. The red dashed lines are the 90% interval and median of the prediction corrected observations. The red shaded area is the 95%‐confidence interval (CI) of the median prediction, blue shaded area is the 95%‐CI of the 5th and 95th prediction interval

The goodness‐of‐fit (gof) plots show no obvious deviations in the plots of CWRES versus time. The η‐shrinkage values on Cl, Q_3_, and V_3_ for the final model were 28%, 9%, and 20% respectively. The ε‐shrinkage for the proportional error model is 13%. The gof plots are shown in Figure 3. The pcVPC shows that the prediction corrected concentrations correspond with the 90% prediction intervals of the 5th, 50th, and 95th percentiles of the prediction corrected simulated concentrations, calculated from 1100 simulated datasets as shown in Figure 4. The stability and performance of the final model was validated using 1100 replicates generated from the original dataset. The mean values of the original dataset were within the 95% CI of the bootstrap values, indicating that all pharmacokinetic parameters can be estimated with acceptable precision (Table 2).

## 4 DISCUSSION

The influence of genetic polymorphisms and other covariates which can affect the disposition of docetaxel was assessed using a PopPK approach. Even though docetaxel is dosed based on BSA, there was a negligible effect of BSA on docetaxel disposition. This is because BSA‐based dosing strategy is advisable for drugs confined to blood volume, dictated by the relationship between body size and blood volume.^15^ Other covariates including age, sex, and creatine Cl did not significantly affect the kinetics of docetaxel which is consistent with the observations made in other studies.^1^, ^16^

Docetaxel is primarily cleared through hepatic metabolism. We observed that patients with elevated ALT had significantly lower Cl of docetaxel. However, only 7/48 (15%) patients had elevated ALT and thus the covariate failed to show statistical significance in the backward elimination step. Thus, ALT was excluded in the final model. Despite this, the influence of liver dysfunction on docetaxel disposition cannot be ruled out. Several studies in the past have demonstrated that hepatic impairment significantly affects the Cl of docetaxel and our findings are consistent with this observation.^17^, ^18^, ^19^ Minami et al. proposed an approximate 20%–40% dose reduction for patients with grade 2 and grade 3 elevation of transaminases at baseline.^17^ In a prospective study by Syn et al. there was noted a 28% and 50% reduction in median Cl in patients with mild and moderate hepatic impairment respectively.^20^

Interestingly, the docetaxel regimen used in various populations is highly variable.^21^ In Caucasians, the routinely used starting doses are 100 mg/m^2^, while in the Asian continent, it is 75 mg/m^2^. This is in line with our study, in which 44 patients have received a dose of 75 mg/m^2^, and 4 patients received 50 mg/m^2^. A possible explanation for the inter‐ethnic difference in docetaxel tolerance lies with the Cl among races.^4^ The population mean estimates of Cl and V_1_ in our study based on the three‐compartment model were 18 L/h and 5.8 L respectively, and the IIV on Cl was 45%. The Cl observed in our study is clearly lower than the reported literature values. The reported Cl of docetaxel ranges from 29.4 to 41.8 L/h.^18^, ^22^, ^23^ Goh et al., showed that docetaxel Cl is approximately 40% lower in Asians, while drug exposure or area under the concentration‐time curve is almost 25% higher, as compared to Caucasians.^22^

ABCB1, ABCC2, and SLCO1B1 are primary transporters of docetaxel. ABCB1 is found to play a crucial role in intestinal absorption and biliary excretion of docetaxel.^23^, ^24^ However, none of the polymorphisms influenced the Cl of docetaxel in our study. The study by Bosch et al. showed a 25% reduction in the docetaxel Cl in homozygous patients with C1236T polymorphism in the ABCB1 gene. The limitations of this study were that of the 92 patients available for PK/PD analysis, pharmacogenetic data were available for only 15 patients, and the rest were obtained from multiple imputations. Goh et al., could not detect any effect of polymorphisms C3435T in ABCB1, CYP3A4*1B, and CYP3A5*3 on the Cl of docetaxel, in an Asian population of 31 NSCLC patients.^22^ Thus, the effects of pharmacogenetic factors on docetaxel Cl are inconsistent and inconclusive among published reports.^8^ Our study is one of the largest to investigate the covariate effect of genetic polymorphisms on docetaxel kinetics. Clearly, the absence of a covariate effect obviates the need for dose individualization of docetaxel based on genetics.

Patients with organ dysfunction are generally not included in clinical trials. This results in scarcity of information related to posology for this group of patients. The current study could not establish the role of hepatic impairment on Cl due to the lack of an adequate number of patients with altered liver function. However, the data in limited number of patients corroborate the findings from previous studies notably by Syn et al. and Minami et al. who recommended 20%–54% reduction in the dose of docetaxel in patients with mild to moderate hepatic impairment based on up to 50% lower Cl observed in such patients.^17^, ^20^ The other covariates such as age, sex, BSA, Alb, creatinine Cl could not significantly explain the IIV to recommend a dose modification for docetaxel.

To conclude, our study characterizes the PopPK parameters of docetaxel in Indian patients. We could not establish a basis for individualizing the dose of docetaxel based on genetic polymorphisms in the ADME genes.

## CONFLICT OF INTEREST

The authors have no conflict of interest.

## Data Availability

The datasets used and analyzed during the current study are available from the corresponding author on reasonable request.
